# Old drugs, new challenges: reassigning drugs for cancer therapies

**DOI:** 10.1186/s11658-025-00710-0

**Published:** 2025-03-05

**Authors:** Paulina Czechowicz, Anna Więch-Walów, Jakub Sławski, James F. Collawn, Rafal Bartoszewski

**Affiliations:** 1 Department of Biophysics, Faculty of Biotechnology, University of Wroclaw, F. Joliot-Curie 14a Street, 50-383 Wroclaw, Poland; 2 Department of Cell, Developmental and Integrative Biology, University of Alabama at Birmingham, Birmingham, USA

**Keywords:** Drug discovery, Drug repurposing, Cancer, Off-label use, Pharmaceutical development

## Abstract

The "War on Cancer" began with the National Cancer Act of 1971 and despite more than 50 years of effort and numerous successes, there still remains much more work to be done. The major challenge remains the complexity and intrinsic polygenicity of neoplastic diseases. Furthermore, the safety of the antitumor therapies still remains a concern given their often off-target effects. Although the amount of money invested in research and development required to introduce a novel FDA-approved drug has continuously increased, the likelihood for a new cancer drug’s approval remains limited. One interesting alternative approach, however, is the idea of repurposing of old drugs, which is both faster and less costly than developing new drugs. Repurposed drugs have the potential to address the shortage of new drugs with the added benefit that the safety concerns are already established. That being said, their interactions with other new drugs in combination therapies, however, should be tested. In this review, we discuss the history of repurposed drugs, some successes and failures, as well as the multiple challenges and obstacles that need to be addressed in order to enhance repurposed drugs’ potential for new cancer therapies.

## Introduction

Despite decades of ongoing efforts in the war against cancer, current therapeutic options often remain insufficient [1, 2]. The unresolved challenge is the complexity and intrinsic polygenicity of neoplastic diseases, which cannot be addressed by targeting a single molecular target. A combination of different treatment modalities is usually required to achieve optimal outcomes that often depend on the individual patient and cancer type. The development of effective and sophisticated treatment strategies is currently fueled by rapid progress in basic biomedical research as well as the evolution of surgical, radiotherapeutic, and immunological approaches.

Nevertheless, the safe application of antitumor drugs is limited by the off-target effects of most current drug strategies. Additionally, their prolonged use can result in modified tumor occurrences and resistance, as well as their ability to promote cancer stem cells [3]. Furthermore, with the acknowledgment of inter- and intra-tumoral heterogeneity and the dynamic and complex interactions within the tumor microenvironment (TME) [4–7], the necessity of simultaneously targeting a variety of different and complex signaling pathways has becomes evident. Consequently, complex combination or multimodal therapies are required [8]. Such approaches could enhance outcomes through their synergistic mechanisms of action by targeting the different properties of cancer cells or the TME. This creates a demand for alternative approaches in drug development [9, 10].

The amount of money invested in research and development (R&D) required to introduce a novel FDA-approved drug is continuously and dramatically increasing, while the likelihood for cancer drug approval in phase I clinical trials remains the lowest of any drug type at 6.7% [10–12]. This limitation in R&D productivity is termed as ‘Eroom’s Law’ [11]. Scannell et al. proposed four problems: (1) ‘the Beatles' problem’. In other words, developing new drugs that were significantly more efficient, safer, and improved over existing (and usually cheaper) therapies was like finding a music group better than the Beatles [13]; (2) the 'cautious regulator' problem is the increasing safety and formal requirements by the regulatory agencies [14–17]; (3) the 'throw money at it' problem occurs when the investment strategies try to overrate a potential new drug in order to improve the company competitiveness [18]; (4) and finally the 'basic research–brute force' bias that improvement of basic research and screening technology will always translate into effective drug discovery [18].

These limitations cannot be easily solved or controlled. Other challenges, however, are being addressed through the development of in vitro pharmacological profiling of drug candidates [19] and the integration of increasing basic research information into drug development pipeline design. This is particularly true in the human genome-wide association studies (GWASs) [20]. Furthermore, the careful consideration of the risk–benefit balance by regulatory agencies is also clearly important [21].

One strategy drug development limitations is the repurposing of already existing, clinically approved drugs for combinatory anticancer treatments [22–24]. This approach allows for the expedited development of novel therapies at a fraction of the costs and risks associated with novel drug discovery given that the safety profiles of these molecules have already been established. Furthermore, drug repurposing strategies are supported by progress in understanding both the mechanisms of human pathologies and the long-term consequences of these “old” drugs’ applications and by the continuous advancements in targeted drug delivery. Notably, the majority of currently marketed drugs have the ability to interact with more than one target, and occasionally include those that could benefit cancer patients [25–27].

Indeed, opportunistic findings led to the very first chemotherapeutics, chlorambucil (Leukeran) and busulfan (Myleran), which are still used to treat chronic lymphocytic and myeloid leukemias (CLL and CML) [28–30]. These drugs originated from ‘mustard gas’ and could be regarded as repurposed chemical weapons [31–33]. Nowadays, repurposing strategies often aim to substitute cytotoxic therapeutics with cytostatic ones, such as metformin and thalidomide, originally used to treat diabetes and inflammation, respectively [33]. Drug repurposing is not only limited to finding new uses for drugs initially developed for other diseases (including generics, on-patent drugs, and failed molecules), but also includes the original cancer drugs reassigned for different types of cancer or in different combinations.

Repurposing drugs can also be faster and less costly. For example, despite the need to test the new indications of repurposed drugs in later-phase clinical trials and obtaining approvals, knowing their safety profiles and pharmacokinetics/pharmacodynamics reduces the risk of failure [34, 35]. Although, the vast majority of molecules that undergo clinical trials do not make it to the clinic [36], some of those that fail at late stages could still be good candidates for repurposing and turn financial losses into potential successes.

In summary, drug repurposing strategies could improve the outcomes of modern mono and combinatory antitumor therapies. However, transitioning repurposed drugs into clinical application is accompanied by numerous commercial, technological, and regulatory obstacles. This short narrative review summarizes and discusses both the advantages and challenges associated with the repurposed drug component of antitumor therapeutic strategies.

### Strategies for drug repurposing

Drug repurposing and is also known as drug repositioning or drug reprofiling. This classification can be further expanded to include failed drugs—compounds that have entered clinical trials but did not succeed due to unsatisfactory efficacy against the initial indication [37, 38]. All these compounds can be further divided into patent-protected (both approved and failed) and off-patent (generic) drugs. Obviously, the majority of research information comes from the latter group [37, 39, 40].

Although many successful drug reassignments have had rather serendipitous backgrounds, these favorable outcomes drive the optimistic and further development of systematic, dedicated strategies [41]. The simplest classification of drug discovery, including repurposing aspects, is divided into two basic lines of action: target-based drug discovery and phenotypic drug discovery [29]. The first approach starts by defining the molecular target underlying the pathology and aims to design dedicated drugs. For example, tamoxifen, which was initially aimed to be a contraceptive, was repurposed for the treatment of breast cancer since it was found to efficiently inhibit the estrogen receptor [42]. In contrast, phenotypic drug discovery ignores a specific drug target or hypothesis about its role in disease and instead tests candidates for desirable biological activity in ‘physiologically relevant’ systems. For instance, during a phenotypic screen for cell proliferation, it was found that auranofin, originally an anti-arthritic medication, effectively and selectively targets gastrointestinal stromal tumors, including imatinib-resistant ones [43, 44].

Drug repurposing strategies can also be classified into three main groups: target-centric, drug-centric, and disease-centric approaches [23]. Each of these strategies has its success stories, limitations, and advantages, and consequently, a dedicated group of supporters. The drug-centric approach focuses on identifying new indications for existing drugs, which can include expanding the existing license or patent towards novel off-label use of the compound in new medical conditions or groups of patients. This strategy is often applied to investigational or failed drugs that faced Eroom’s Law-related limitations in their initial assignment pipelines [23]. For example, valproic acid, originally indicated for bipolar disorder, has an off-target interaction with histone deacetylase 2, a protein that plays a role in many types of cancers. This has led to testing the repositioning of this drug for the treatment of neoplastic conditions such as familial adenomatous polyposis [45].

Drug-centric repositioning can be considered the least direct approach because the drug is only linked to a novel indication via the discovery of a target that is already established for this indication. Thus, a precise characterization of drug-target interactions is required to propose a novel repositioning hypothesis. The most common technical approaches for drug-centric repositioning are structure-based computational methods like molecular docking [46], pharmacophore modeling algorithms [47], protein–ligand interaction profile similarity testing [48], and machine learning approaches [49, 50]. However, since the drug needs to be repurposed to a novel target or disease, a structural model describing the binding mode of the drug to its original targets needs to be well defined. Furthermore, machine learning approaches are limited by the completeness of information in the databases that are utilized [20, 23, 51].

The target-centric approach matches a new indication without a treatment with an established drug and its known target. The old and new indications typically differ quite significantly. Complementary to a disease-centric approach, target-centric repositioning builds on a novel link between a new indication and an established target. It involves investigating the specific molecular targets implicated in the pathology of a disease and uses an existing drug proven to modulate those targets. For example, azacitidine, a potent inhibitor of DNA methyltransferases, was originally dedicated to treating myelodysplastic syndrome [52] and was later adapted for treating patients with acute myeloid leukemia and chronic myelomonocytic leukemia [52]. This approach is particularly useful when seeking to repurpose drugs to treat rare diseases.

Finally, the most effective approach so far is the disease-centric approach, which involves re-profiling drugs among different types of a disease, such as two types of cancer [23]. It involves identifying diseases with homologous underlying biological mechanisms and similar guiding principles to the indicated original drug treatments. For example, a drug developed to treat psoriasis could also treat other diseases with uncontrolled cell growth, such as cancer. In the case of cancer, these guiding principles are summarized in the Hallmarks of Cancer [53, 54]. Since these key hallmarks of malignancy are not regulated by a single signaling pathway [55], the pathways responsible for a cancer phenotype underlie the pathomechanisms of many other non-oncological human diseases. This opens the possibility of repurposing drugs towards novel anticancer therapeutics and supports agents in combinational approaches [56]. Notably, the diversity and complexity of the hallmarks of oncogenesis provide a strong rationale for using multiple drugs to obtain satisfactory therapy outcomes [40], while drug repositioning could significantly reduce costs and increase the availability of such novel therapies [56, 57].

### Cancer complexity as a target of repurposed drugs

With the progress in understanding the molecular mechanisms related to oncogenesis, cancer progression, and treatments, more distinct attributes and signaling pathways are now recognized as crucial for various neoplastic diseases. Indeed, the initially proposed eight Hallmarks of Cancer have now been extended to fourteen [53, 54]. Although this diversity and complexity of neoplastic disease remain a therapeutic challenge, it also provides a strong rationale for drug repurposing. It is important to note that these hallmarks often result from the crosstalk between different signaling pathways, some of which are deregulated in cancer cells, while others compensate for this [58–62]. For example, cancer cells often efficiently avoid unfolded protein responses, hypoxia, and/or oxidative stress-related cell death signals and favor the proadaptive ones [63–67].

Nevertheless, the complexity of such signaling often limits the ability to selectively target cancer-specific signals without interfering those that are therapeutically desired. For example, inositol-requiring enzyme 1 (IRE1) activity, which is of great interest as a target for glioblastoma and triple-negative breast cancer, can lead to the accumulation of proadaptive signals. Whereas, at the same time, this enzyme can also support cell death signaling [7, 68–70]. Thus, despite the availability of both IRE1 inhibitors and potentiators, their translation into the clinic remains a challenge. Furthermore, healthy cells are often exposed to stress-inducing factors, with chemotherapeutics being one of them [71], and thus such stress-oriented therapies also carry the risks of adverse effects.

Chronic proliferation, a fundamental feature of cancer cells, often results from a network of deregulated signaling pathways and growth factors. These pathways operate mainly by receiving signals from growth factors that bind to cell-surface receptors typically with intracellular tyrosine kinase activity [72]. This activity modulates pro-survival signaling pathways such as phosphatidylinositol 3-kinase/protein kinase B (PI3K/AKT), the mammalian target of rapamycin (mTOR) [73] and the mitogen-activated protein kinases/extracellular signal-regulated kinase (MAPK/ERK) [74]. The development of specific receptor tyrosine kinase inhibitors has resulted in effective chemotherapeutics [74]. However, cancer cells can also utilize many other pathways to proliferate and eventually circumvent the inhibited routes in order to develop resistance [75, 76]. Repurposed drugs may provide a solution to this problem. For example, rapamycin, an mTOR1 inhibitor initially approved as an immunosuppressant and later as an anti-restenosis agent [77, 78], was repurposed to treat leukemias due to the importance of the mTOR pathway in cancer [79, 80] [81]. However, inhibition of mTOR1 is often compensated by the activation of PI3K-AKT [82] and the reactivation of eukaryotic translation initiation factor 4E-binding proteins [83, 84]. Despite these limitations, rapamycin and its more soluble and specific analogs, like temsirolimus [85], have been tested in combination with growth factor receptor antagonists [86].

Other candidates for repurposing that could target cancer proliferation include prazosin, an alpha blocker initially approved to treat hypertension [87], and indomethacin, a non-steroidal anti-inflammatory drug (NSAID) [88]. Prazosin has been reported to inhibit AKT signaling [89, 90] and is recommended for treating pheochromocytoma [91]. It is also included in a Phase 1 study as an additive to radiotherapy in men with prostate cancer (ACTRN12621000784819). Indomethacin, besides its Cox1/2 inhibition-related antiangiogenic effects [92], has been shown to impair cancer proliferation by inhibiting MAPK [93] or PKC signaling [94]. Currently, three indomethacin-related clinical trials are registered, including a Phase 4 trial for prostate cancer (ChiCTR2000038968) and a Phase 1 study for breast cancer (NCT02950259). Furthermore, drugs that could effectively target human telomerase reverse transcriptase (hTERT) in cancer cells to limit their replication remain an interest for drug repurposing strategies [95].

Another promising strategy is the search for drugs that can accelerate cancer cell death. Along with progress in understanding the molecular mechanisms underlying regulated cell death [96, 97], many related adverse effects of non-oncological drugs may be useful in cancer therapies. Considering that cancer cells often circumvent common apoptotic pathways, drugs that can selectively accelerate other cell death modalities—including ferroptosis [98], cuproptosis [99], necroptosis [100], pyroptosis[101], and lethal autophagy [102]—can improve treatment strategies while reducing general toxicity. One exception, however, is autophagy since this can also favor cancer cells by enhancing their survival under metabolic and environmental stresses [103], and therefore should be considered with caution.

Artemisinin and chloroquine, along with their derivatives were initially dedicated for treating malaria [104], are well-known examples of cell death-related drug repurposing. Artemisinins have been reported to induce non-apoptotic programmed cell death, especially ferroptosis, in cancer cells [105–109]. Recent clinical trials for artesunate are testing its application in colorectal cancers (NCT02633098) and leukemias (CTRI/2024/03/063617). Chloroquine and its derivative hydroxychloroquine are approved as autophagy flux inhibitors to treat pancreatic and other cancers [110, 111]. Currently, these compounds are undergoing 48 clinical trials related to cancer therapies (Database Repurposing Trials In Oncology, ReDO_Trials_DB, https://www.anticancerfund.org/en/database-repurposing-trials-oncology as for 14.08.2024) [112].

Mebendazole (5-benzoyl-1H-benzimidazol-2-ylcarbamate), initially intended as an anthelmintic agent [113], is another candidate for repurposing into oncological therapies due to its potential to inhibit microtubule polymerization [114, 115]. Notably, besides restricting tumor growth, this compound has been effective in preventing the invasion and metastasis of malignant tumors and glioblastomas [116, 117]. Zhang [118] and in other individual cancer cases [114, 115]. Currently, six mebendazole-related clinical trials are registered in the ReDO_Trials_DB [112].

Deregulated metabolism and increased energy demands are other hallmarks of cancer [119, 120] that have been targeted for drug repurposing [121]. Patients with diabetes are generally more prone to several types of cancer [122, 123]. Long-term treatment with metformin, approved for obese type 2 diabetes [124], has been observed to lower the risk of cancer in diabetic patients, making this drug a potential candidate for repurposing [120, 122]. Indeed, there are 133 metformin-related trials in the ReDO_Trials_DB [112]. However, despite these efforts, results have been far from satisfactory [125, 126]. Furthermore, the mechanism of action of metformin in tumor cells and the tumor microenvironment remains unclear and under discussion. Although high doses of this compound impair cellular respiration by inhibiting Complex-1 [127], preclinical studies have observed a plethora of pleiotropic effects of metformin administration in cancer cells that are independent of Complex-1 inhibition [128, 129]. Hopefully, continuous research on this compound will eventually allow successful metformin repurposing.

Disulfiram provides another example of a repurposed drug, initially intended to treat alcoholism [130], now targeting cancer cell metabolism ^129,130^. Disulfiram inhibits acetaldehyde dehydrogenase activity [131], resulting in alcohol intolerance, as well as the blockage of formaldehyde oxidation [132] and deregulated oxidative metabolism in cancer cells [133]. Furthermore, the p97 segregase adaptor NPL4 (also known as VCP), important for maintaining cellular proteostasis, has been identified as the molecular target of disulfiram responsible for its anticancer activity [134]. Currently, there are 13 disulfiram clinical trials in the ReDO_Trials_DB, including a phase 2/3 application of this drug for glioblastoma (NCT02678975) [112].

The ability of cancers to impair and circumvent host immune responses provides another therapeutic opportunity [135–138]. Pharmaceutical solutions that could increase antitumor immunity, such as immune checkpoint inhibitors, are currently of great interest [139]. It has been observed that some vaccines dedicated to infectious diseases (such as rotaviruses, yellow fever, and influenza), when administered intratumorally, can activate antitumor immunity [140–143].

Other drug repurposing strategies arise from the specific impairment of tumor suppressors such as p53 or the retinoblastoma protein in cancer cells [144]. Along these lines, quinacrine, an antimalarial agent [145], was found to be a promising candidate since it was reported to induce p53 expression in cancer cells [146] and can exert some anticancer activity in a p53-dependent manner [146, 147]. The accumulation of p53 has also been observed in cancer cells treated with ritonavir, a protease inhibitor used to treat human immunodeficiency virus (HIV) infection [148, 149]. Furthermore, other reports found this compound capable of reactivating the retinoblastoma protein [147]. Currently, there is one active phase 1 clinical trial for the application of ritonavir for prostate cancer (NCT05679388) [112]. Notably, statins have also been shown to increase p53 activity in cancer cells and thus display anticancer potential [148, 149]. Currently, there are 47 statin-related records in the ReDO_Trials_DB, many of which are reaching phase 3 or 4 [112].

Tumor expansion is accompanied by an increased demand for nutrients and oxygen by cancer cells, leading to the induction of chronic angiogenesis [150]. Although this hallmark of cancer cells has resulted in the development of currently used antiangiogenic agents that limit tumor blood flow and lead to its starvation [58, 151], the use of these drugs is limited and can unfortunately stimulate resistance [152]. Notably, the repurposing of thalidomide, an immunomodulatory drug initially sold to treat morning sickness that was withdrawn worldwide in 1962 after it was linked to severe birth defects [153], has shown promise. Thalidomide still remains in use to treat leprosy [153] and is currently used as an antiangiogenic agent [154, 155]. It is also approved for combination therapy in multiple myeloma [156].

Antiangiogenic and anticancer potential has also been observed for the approved antifungal agent itraconazole [157, 158]. Currently, there are 17 itraconazole-related records in the ReDO_Trials_DB, one of which for ovarian cancer is reaching phase 3 (NCT03458221). Interestingly, artemisinins have also been assigned antiangiogenic activities [159]. In contrast, it has been reported that some anticancer approaches aim to induce angiogenesis in order to facilitate drugs delivery [160, 161].

Notably, cancer-related inflammation supports not only angiogenesis but also invasion and metastasis, as well as reprogramming of the tumor microenvironment (TME) [162, 163]. Therefore, anti-inflammatory drugs could be good candidates for repurposing. Celecoxib, a selective cyclooxygenase-2 (COX-2) inhibitor approved for adult arthritis [164], is extensively being tested in clinical trials (57 registered in ReDO_Trials_DB) due to multiple reports of its potential to enhance the chemosensitivity of cancer cells and to reduce the toxicity of marketed chemotherapeutics [165–167]. Similarly, 31 clinical trials are registered for aspirin (ReDO_Trials_DB), which was already suggested to have potential as an anticancer drug in the 1970s [168]. More recent research reports of aspirin being effective against many types of cancer [169–171] are supported by epidemiological analyses [172–175]. Indeed, low-dose aspirin inhibits the production of thromboxane A2 (TXA2) by irreversibly inhibiting the enzyme COX-1 in platelets. By reducing TXA2, aspirin can help prevent the formation of blood clots and has been shown to have potential benefits in reducing the risk of cancer progression and metastasis [176, 177]. Furthermore, aspirin at higher doses is also a more potent COX-2 inhibitor, which can increase its anti-cancer properties against tumors that overexpress this enzyme [175]. Numerous studies shown that daily low-doses of aspirin may significantly reduce the risk of colon cancer and rectal cancer [178] and breast cancer [179, 180]. Recent results from a 20-year cohort study involving 1,909,531 individuals in Denmark have shown that long-term low-dose aspirin use is associated with slightly to moderately reduced risks for several specific cancers [181]. However, there was no reduction in overall cancer risk for some common cancers [181]. Similar or slightly stronger inverse associations were observed for the consistent use of high-dose aspirin [181].

Interestingly, the ASPirin in Reducing Events in the Elderly (ASPREE) study, which was double-blind and performed on a large cohort (for 4.7 years and over 19,000 individuals older than 65–70, that did not have cardiovascular disease, dementia, or disability), showed no advantage of taking low-dose aspirin, and in fact increased the risk of being diagnosed with stage 3 or 4 cancers as well as increased mortality rates compared to the placebo [182]. In contrast, prior randomized controlled trials, mainly involving younger individuals, demonstrated a delayed cancer benefit with aspirin [182]. However, these study conclusions are under discussion and should be taken with caution, as deaths were classified according to the underlying cause by adjudicators who were unaware of the trial-group assignments. Furthermore, hazard ratios were calculated to compare mortality between the aspirin group and the placebo group, and post hoc exploratory analyses of specific causes of death were performed [182].

Furthermore, some of the adverse effects of many approved chemotherapeutics can be reduced with the use of additional anti-inflammatory compounds and beta-blockers to reduce cardiotoxicity [183, 184]. The latter (especially propranolol and timolol) have been reported to have anticancer activity [185–187]. Taken together, these examples provided illustrate the vast potential of drug repurposing in oncology.

## Discussion

**Fig. 1:**
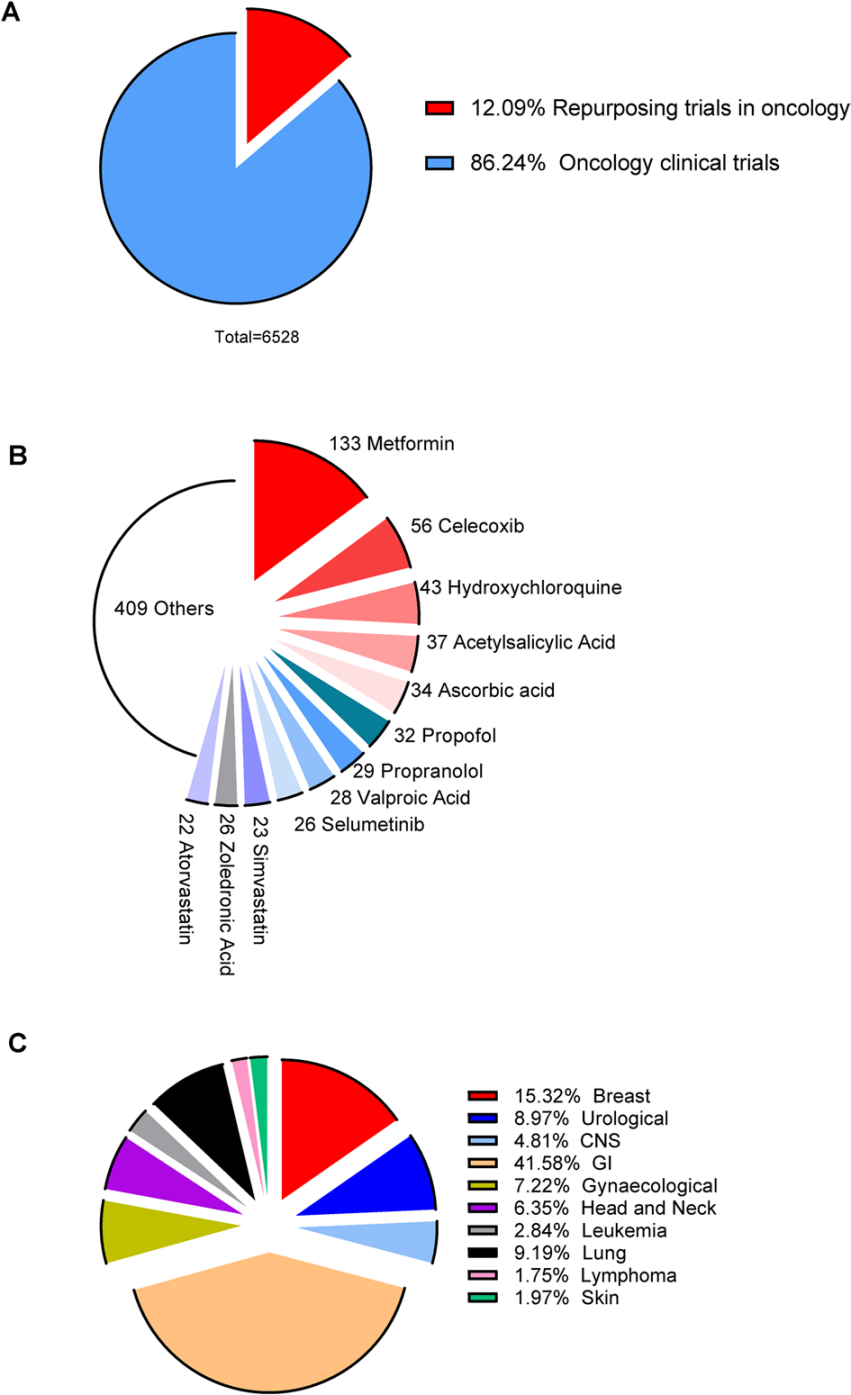
**A**. Repurposing trials are a modest percentage of all oncology clinical trials. **B** The most popular drugs in repurposing trials (**C**) and the most popular cancers to be targeted in controlled trails of repurposed drugs. The data were obtained from the ReDO_Trials_DB database and the GlobalData’s Clinical Trials Intelligence Center (December 15, 2023)

Although the drug repurposing approach seems like an attractive solution to benefit both cancer patients and health systems, its results have been below expectations despite the considerable efforts and high academic attention. Continuous research and technological development have yet to yield breakthrough solutions. While there are some examples of successful drug repositioning, the majority of candidates, like metformin, have remained in the reassignment pipelines for many years. As of August 14, 2024, the ReDO_Trials_DB database reports 898 (409 controlled) trials related to 182 drugs (with metformin being the most commonly tested one) and involving 157,295 patients [112] (Fig. 1). Notably, however, less than 5% of these trials are sponsored by pharmaceutical companies, with the vast majority funded by non-profit organizations [188, 189]. Furthermore, 139 clinical trials have reached phase 3 or 4 [112].

Although these numbers may initially seem impressive, they are modest compared to data from the GlobalData’s Clinical Trials Intelligence Center ((https://www.clinicaltrialsarena.com/sponsored/oncology-in-2024-the-clinical-trial-trends-reshaping-the-role-of-cros/). As of December 15, 2023, 6,528 oncology-related trials were noted, with another 569 planned to begin. In the DrugBank database ((https://go.drugbank.com/releases/latest), there are 4,493 approved drugs (2812 small molecules and 1681 biotechnology drugs) that can be directed against over 3,000 unique targets (Fig. 1). Additionally, there are over 333 withdrawn drugs (302 small molecules and 31 biotechnology drugs), 8,000 investigational ones (5311 small molecules and 2752 biotechnology drugs), and about 6732 experimental compounds (6353 small molecules and 379 biotechnology drugs). Taken together, drug repurposing in oncology, despite its potential and large number of possible candidates, remains underappreciated and mostly limited to academic research and small biotech companies.

Notably, the business models of Big Pharma rely strongly on market exclusivity for their drugs, allowing them to sell their drugs at high prices [190–192]. Since repurposed drugs cannot usually be considered novel chemical entities and their structures are already known, novel patent claims to the active pharmaceutical ingredient are not possible [193]. The repurposed drug can only be protected at the level of the ‘method of use’ for the new indication, although such protection is harder to obtain and costs more [193]. Finally, the use of patents excludes off-label prescriptions, where medications are prescribed for indications or populations for which they have no regulatory approval [194]. Numerous solutions have been suggested to motivate Big Pharma efforts towards repurposing (tax breaks, FDA-priority review vouchers, or funding clinical trials) [195, 196]. However, given the current business model Big Pharma operates under, implementing these initiatives probably will not be a game changer [188]. This conflict of interest is well illustrated by thalidomide approvals. Despite this drug in combination with melphalan–prednisone is comparable to the dramatically more expensive lenalidomide, the more expensive new drug was approved as the standard therapy [197, 198].

Indeed, mainly academic and independent research provides the rationale for using off-patent medications in cancer treatment [40]. However, in the case of drug repurposing, these academic approaches often lack specific insights that are exclusive to pharmaceutical companies. Due to limited resources, lack of data, technology, funding, and experience, many academic attempts at drug repurposing are often “fashion” driven (metformin, statins, aspirin, ascorbic acid, etc.) [112] and thus oriented on drugs that may be easy to obtain, publish, and get funded for their application (Fig. 1B). However, this approach does not consider that millions of people who are cancer patients or will develop cancers are already taking these prescriptions, whereas their benefits on a wide population scale in terms of cancer risks are usually under discussion or not properly documented.

Furthermore, academic research is the main contributor of related omics and epidemiological data that are further used for machine learning and other applications utilized by drug repositioning strategies. Notably, retrospective observational studies are subject to immortal time bias and selection bias [199], resulting in frequent overestimations of their advantages for the treatment group [200]. This case is well illustrated by the numerous correlations between metformin treatments and the incidence of cancer [201]. Furthermore, since metformin’s original target group were diabetic patients who differ from cancer patients, selection bias occurred [202]. Additionally, computational strategies for predicting drug reassignment are only as good as the reliability of the input data and can often discourage target-centric and drug-centric strategies. Phenotype-based high throughput screening strategies, due to the costs of libraries and extensive labor, are usually beyond basic research funding schemes. Finally, the costs and ability to design dedicated and sufficient clinical trials that can include various cancer types and patient groups, remain another serious barrier for academic approaches.

The complexity of cancer limits the efficacy of monotherapies, whereas combination therapies come with specific challenges and limitations that often translate into drug repurposing approaches [199, 203, 204]. Notably, combination therapy trials are more complex and thus cost more than those for monotherapies [205]. Since phase 2 trials are usually the turning point for a drug’s fate, progressing them towards more randomized larger cohort research trials requires promising efficacy results in order to justify the financial burden [199].

Many clinical trials fail due to insufficient cancer patient accrual [206]. Cancer patients are often older people with accompanying diseases and undergoing different cancer-related or unrelated treatments, which may result in unexpected side effects and death during the trials [33, 207]. Indeed, combinational therapies do not guarantee better efficacy [33]. For example, combined dacarbazine with cisplatin, carmustine, and tamoxifen for metastatic (stage IV) melanoma treatment is comparable in terms of patient survival with monotherapy based on high-dose dacarbazine [207, 208]. Unfortunately, dosages that may be well tolerated in trials conducted using healthy subjects or when used for treating the diseases the drug was originally intended for may not be achievable in cancer patients [33]. Repurposed drugs intended for specific targets might not have the same efficacy in cancer cells or in the presence of other drugs [33]. Therefore, their repositioning may require higher doses, which can result in novel distinct mechanisms of action and consequently unforeseen adverse effects [209, 210]. For example, aspirin repurposed for use in high doses may lead to an increased risk of gastrointestinal bleeding, while simvastatin and metformin contribute to the development of hypolipidemia and hypoglycemia. All these issues stress the importance of quality preclinical research that will allow only the most effective and safest combinations to be trialed in phases 2 and 3 [204, 211, 212].

Importantly, recent scientific and technological breakthroughs are becoming more economically available and thus possible to incorporate into high-throughput screening pipelines, as well as drug-centric approaches. Starting with omics approaches, which allow the determination of molecular mechanisms of repurposing candidates, and coupling this with failed drugs could provide valuable insight into the cellular proteomic, metabolomic, and transcriptomic changes [44, 213–222] in various cancers. Importantly, single-cell sequencing seems to be a way to address cancer heterogeneity [223], while the rapid growth and development of databases provide machine learning and computational approaches with more reliable insights [224–227]. Furthermore, the development of novel drug delivery methods that allow more specific and even compartment-targeted application of repurposed drugs may contribute to the success of novel repurposing strategies [228–230].

## Conclusions and perspectives

Repurposed drugs have the undeniable potential to address the shortage of new drugs and combat acquired chemotherapy resistance. Additionally, many healthcare systems struggle to provide patients with expensive new generations of chemotherapeutics, let alone personalized therapies. The financial advantages of drug repositioning could benefit many patients worldwide. However, despite research progress, multiple pharmacological challenges and obstacles need to be addressed to effectively utilize the opportunity of repurposed drugs in cancer treatment. Importantly, the field requires programs, regulations, and government-level funding to promote and support collaboration between academia and the pharmaceutical industry [40, 190]. Such programs in the USA and UK have allowed the transfer of a large number of failed drugs to academic research for repurposing [231]. Furthermore, continuous efforts and lobbying by academic and non-profit organizations are necessary to popularize and develop new drug discovery concepts.

## Data Availability

Not applicable.

## Abbreviations

AKT: Protein kinase B
ASPREE: ASPirin in Reducing Events in the Elderly
CLL: Chronic lymphocytic leukemia
CML: Chronic myeloid leukemia
COX-2: Cyclooxygenase-2
ERK: Extracellular signal-regulated kinase
FDA: Food and Drug Administration
GWASs: Genome-wide association studies
HIV: Human immunodeficiency virus
hTERT: Human telomerase reverse transcriptase
MAPK: Mitogen-activated protein kinases
mTOR: Mammalian target of rapamycin
NSAID: Non-steroidal anti-inflammatory drug
PI3K: Phosphatidylinositol 3-kinase
R&D: Research and development
TME: Tumor microenvironment
